# Interactive reservoir computing for chunking information streams

**DOI:** 10.1371/journal.pcbi.1006400

**Published:** 2018-10-08

**Authors:** Toshitake Asabuki, Naoki Hiratani, Tomoki Fukai

**Affiliations:** 1 Department of Complexity Science and Engineering, Univ. of Tokyo, Kashiwa, Chiba, Japan; 2 Gatsby Computational Neuroscience Unit, Univ. College London, London, United Kingdom; 3 RIKEN Center for Brain Science, Wako, Saitama, Japan; Université Paris Descartes, Centre National de la Recherche Scientifique, FRANCE

## Abstract

Chunking is the process by which frequently repeated segments of temporal inputs are concatenated into single units that are easy to process. Such a process is fundamental to time-series analysis in biological and artificial information processing systems. The brain efficiently acquires chunks from various information streams in an unsupervised manner; however, the underlying mechanisms of this process remain elusive. A widely-adopted statistical method for chunking consists of predicting frequently repeated contiguous elements in an input sequence based on unequal transition probabilities over sequence elements. However, recent experimental findings suggest that the brain is unlikely to adopt this method, as human subjects can chunk sequences with uniform transition probabilities. In this study, we propose a novel conceptual framework to overcome this limitation. In this process, neural networks learn to predict dynamical response patterns to sequence input rather than to directly learn transition patterns. Using a mutually supervising pair of reservoir computing modules, we demonstrate how this mechanism works in chunking sequences of letters or visual images with variable regularity and complexity. In addition, we demonstrate that background noise plays a crucial role in correctly learning chunks in this model. In particular, the model can successfully chunk sequences that conventional statistical approaches fail to chunk due to uniform transition probabilities. In addition, the neural responses of the model exhibit an interesting similarity to those of the basal ganglia observed after motor habit formation.

## Author summary

Varieties of information processing require chunking, but chunking arbitrary complex sequences as flexibly as the brain does remains a challenge. In this study, we solved this important but difficult problem of chunking by "reservoir computing" inferred from brain computation. In the method, chunking occurs automatically when a pair of such modules supervising one another agree on the recurring response patterns to remember. Our model revealed a unique dynamical mechanism for embedding the temporal community structure of inputs into low-dimensional neural trajectories. From a biological viewpoint, our model based on pairwise computing suggests the computational implications of brain's bi-hemispheric information streams. Owing to recent technological advances, the implementation of this model by electronic devices should be straightforward.

## Introduction

When a sequence of stimuli is repeated, they may be segmented into “chunks,” which are then processed and stored as discrete units. This process, called “chunking” or "bracketing" [1], takes place during various cognitive behaviors that require hierarchical sequence processing [2–5]. For instance, in motor learning, a sequence of smaller movements may be executed as one compound movement after repetitive practice [1,6–9]. During language acquisition, continuous vocal sounds are segmented into familiar groups of contiguous sounds that are processed as words [10, 11]. Chunking is believed to reduce the complexity of sequence processing and hence the associated computational cost [1, 12–13]. In this regard, chunking constitutes a crucial step in representing the hierarchical structure of sequential knowledge in neural circuits [14].

Chunking is believed to occur through two consecutive processes. Long and complex sequences are first segmented into shorter and simple sequences, while frequently repeated segments are concatenated into a single unit [15]. Various mechanisms of chunking have been proposed based on Bayesian computation [4, 16], statistical learning guided by prediction errors [17], and a bifurcation structure (stable heteroclinic orbits) in nonlinear dynamical systems [18, 19]. In addition, a neuromorphic hardware has been proposed [20]. However, none of these mechanisms have been shown to chunk with the level of flexibility that the brain offers. It also remains unclear whether a bifurcation theoretic mechanism exists that enables the chunking of arbitrary complex sequences. Many studies evaluating event segmentation in biological and artificial systems have focused on mechanisms to detect boundaries between events by transient increases in surprise signals, which are thought to form based on unequal transition probabilities among sequence elements [4, 14, 21–22]. However, human subjects can segment sequences of visual stimuli that have uniform transition probabilities and hence cannot be chunked by any variation of such mechanisms [23]. These findings suggest that biological neural networks favor a mechanism of chunking that is based on temporal community detection, in which stimuli that frequently go together are grouped into a chunk. A similar mechanism may also account for the brain’s ability to detect repetitions of patterned stimuli in random sequences [24–26].

However, the logic and neural mechanism of flexible and automatic chunking by the brain remain unknown. In this study, we propose a novel mechanism of unsupervised chunk learning based on a unique computational framework that differs from any of the previous proposals. In this mechanism, neural networks learn the low-dimensional dynamical trajectories embedding stereotyped responses to recurring segments (chunks) of a temporal input. We achieve this mechanism in a framework of cortical computation [27, 28] by extending reservoir computing (RC) to unsupervised learning. RC is a high-dimensional dynamical system consisting of a recurrent neural network, readout units, with feedforward and feedback projections between them, and supervised learning in its original form [29]. We were able to attain unsupervised learning in a pair of independent RC modules supervising each other without any external instructive signal. As a consequence, they learned to mimic, or predict, the preferential responses of partner modules to chunks in a given temporal input.

The primary interest of this study was determining the novel computational mechanism to segment information streams. However, an unexpected finding included a surprising similarity between the temporal response patterns of readout units in our model and a functional subtype of basal ganglia neurons, called stop cells, which are observed after habituation [7–9]. This finding suggests that the proposed paradigm of sequence processing has a biological relevance.

## Results

### Reservoir computing modules with mutual supervision

**Fig 1:**
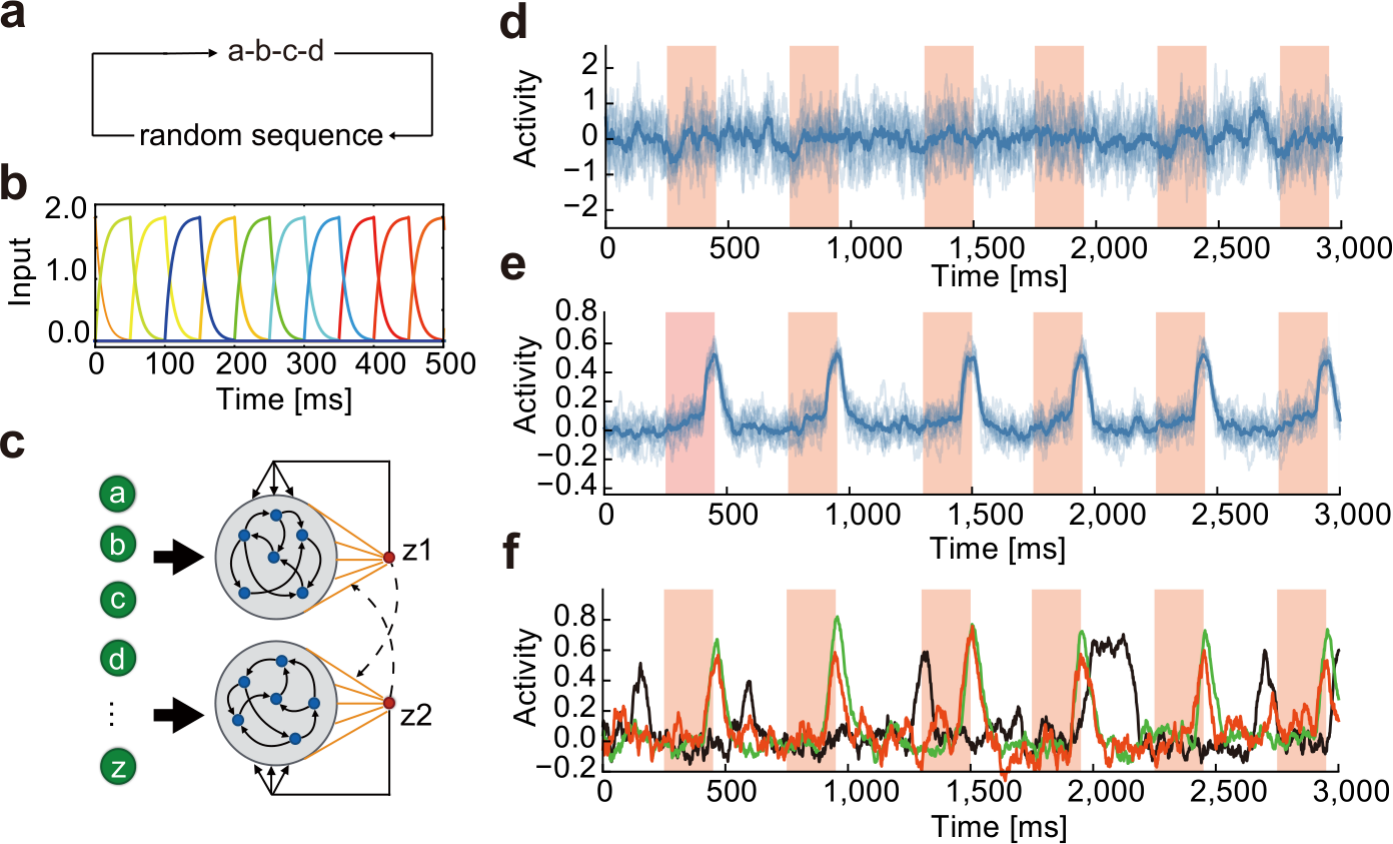
Learning of a single chunk repeated in random sequence. **(a)** Input sequence repeating a single chunk. In this example, the chunk is composed of four alphabets (a, b, c, d). The components and lengths of random sequences varied during the repetition of chunks. **(b)** Example responses are shown for input neurons. **(c)** In the dual RC model, two non-identical reservoirs are activated by the same set of input neurons. Readout weights of each RC system undergo supervised learning with a teaching signal given by the output of the partner network. **(d)** and **(e)** Pre- and post-learning trial averaged activities of a readout unit are shown, respectively. Shaded intervals designate the presentation periods of the chunk. The other readout unit exhibited a similar activity pattern. (**f**) Readout activity was trained with many-to-one input projections. The fraction of input neurons projecting to a reservoir neuron was 10% (red), 40% (green) and 70% (black).

To demonstrate the basic framework of our model, we first consider the case where the input sequence alternates a single chunk (i.e., a-b-c-d) and random sequences of discrete items, which are chosen from the remaining 22 letters of the English alphabet (e to z) (Fig 1A). In reality, each letter may correspond to a brief stimulus in any sensory modality, such as a brief tone signal, and is given to the model through phasic activation of a specific input neuron (*I*_*μ*_(*t*) in Eq 4 in the Methods) with slow rise and decay constants (Fig 1B). Thus, the number of input neurons coincides with that of letters. The random sequence components are introduced to unambiguously define the initial and end points of a chunk, and their lengths vary with every repetition cycle within the length range of 5 to 8.

Our network model comprises two mutually non-interacting RC modules, each of which consists of a recurrent network (reservoir) of rate-based neurons and a readout unit. Each RC module receives an identical input sequence (Fig 1C). Each reservoir neuron receives a selective input from one of the input neurons and hence has a preferred stimulus. As shown later, however, this constraint is not essential for chunking and can be relaxed. Within each reservoir, all neurons are mutually connected and project to a readout unit, which projects back to all neurons belonging to the same reservoir. Note that the two reservoirs have different recurrent wiring patterns and hence are not identical. The activity of each readout unit *z*(*t*) is given as a weighted sum of the activities ***r***(*t*) of the reservoir neurons projecting to the readout: *z*(*t*) = ***w***^T^***r***(*t*). Note that one readout unit per reservoir is sufficient for learning a single chunk. We will consider more complex cases later. The weight vector ***w*** is modifiable through the FORCE learning algorithm [29], whereas the recurrent and feedback connections are non-plastic because the model can solve the present task without modifying these connections. The initial states of the reservoirs are weakly chaotic as in the previous model [29]. See the Methods for details of the model and values of the relevant parameters.

A unique feature of the present model is that the output of each readout unit is used as a teacher signal to train the readout weights of the other reservoir module, implying that the two RC modules supervise each other. As a consequence, although the FORCE learning per se is a supervised learning rule, the entire network, which we call the "dual RC system," is subject to unsupervised leaning because teaching signals originate from the system itself. The details of the teaching signals will be shown later.

### Chunk learning from a random sequence

The design of the teaching signals is the key for successful chunk learning in the present model. The teaching signals should be symmetric with respect to the interchange of the two readout units, and should be determined such that the two systems stop learning when the two readout units output similar response patterns. In other words, the teaching signals eventually become identical between the two RC modules during learning. The following teaching signals *f*_*i*_ enable chunk learning in the proposed dual RC system:

$$f_{i}(t)={\left[tanh({\hat{z}}_{j}(t)/\beta )\right]}_{+.}\;(i,j=1,2;\;i\neq j)$$ (1)

where ${\hat{z}}_{i}$ is the normalized output of the *i*-th readout unit (Methods), the threshold linear function [*x*]_+_ returns 0 if *x*≦0, and [*x*]_+_ = *x* if *x*>0, and the constant was set as *β* = 3. Defining error signals as *e*_*i*_(*t*) = *z*_*i*_(*t*) – *f*_*i*_(*t*), we trained the pair of RC modules through the FORCE learning algorithm until the error signals become sufficiently small (typically, about 0.01) and the readout weights converge to equilibrium values (within small fluctuations). The sigmoidal function allows the system to learn nontrivial solutions *z*_*j*_(*t*) ≠ 0, while maintaining the outputs (and hence the teaching signals) to be finite during learning. Furthermore, the saturation part of sigmoidal function prevents the model from responding too strongly to a specific chunk and makes it easier to detect all the chunks embedded in the input sequence. This activity regulation is particularly important in the learning of multiple chunks studied later. The threshold linear function makes the outputs positive; these nonlinear transformations greatly improved the performance of learning. Importantly, the teaching signals do not explicitly contain information about the structure and timing of chunks in the input sequence. This dual RC system converged to a state of stable operations when the two RC systems produced similar teaching signals (hence similar outputs) that were consistent with the temporal structure of the input sequence (S1 Fig). The readout units did not respond to the chunk before learning (Fig 1D). After learning, the responses of the readout units were tested for the input sequences that had not been used for the training. The test sequences contained the same chunk “a-b-c-d,” but the random sequence part was different. Given these inputs, the readout units exhibited steady phasic responses time-locked to the chunk (Fig 1E). The readout activity piled up gradually in the beginning of the chunk, rapidly increased at its end, and then returned to a baseline level. The selective responses to the chunk were also successfully learned when each reservoir neuron was innervated by multiple input neurons. As shown in Fig 1F, the system succeeded in learning when randomly-chosen 10% or 40% of input neurons projected to each reservoir neuron, but failed when the fraction was 70%. Thus, responses of the individual reservoir neurons should be sufficiently independent of each other to robustly capture the recurrence of chunks.

### Learning of multiple chunks

**Fig 2:**
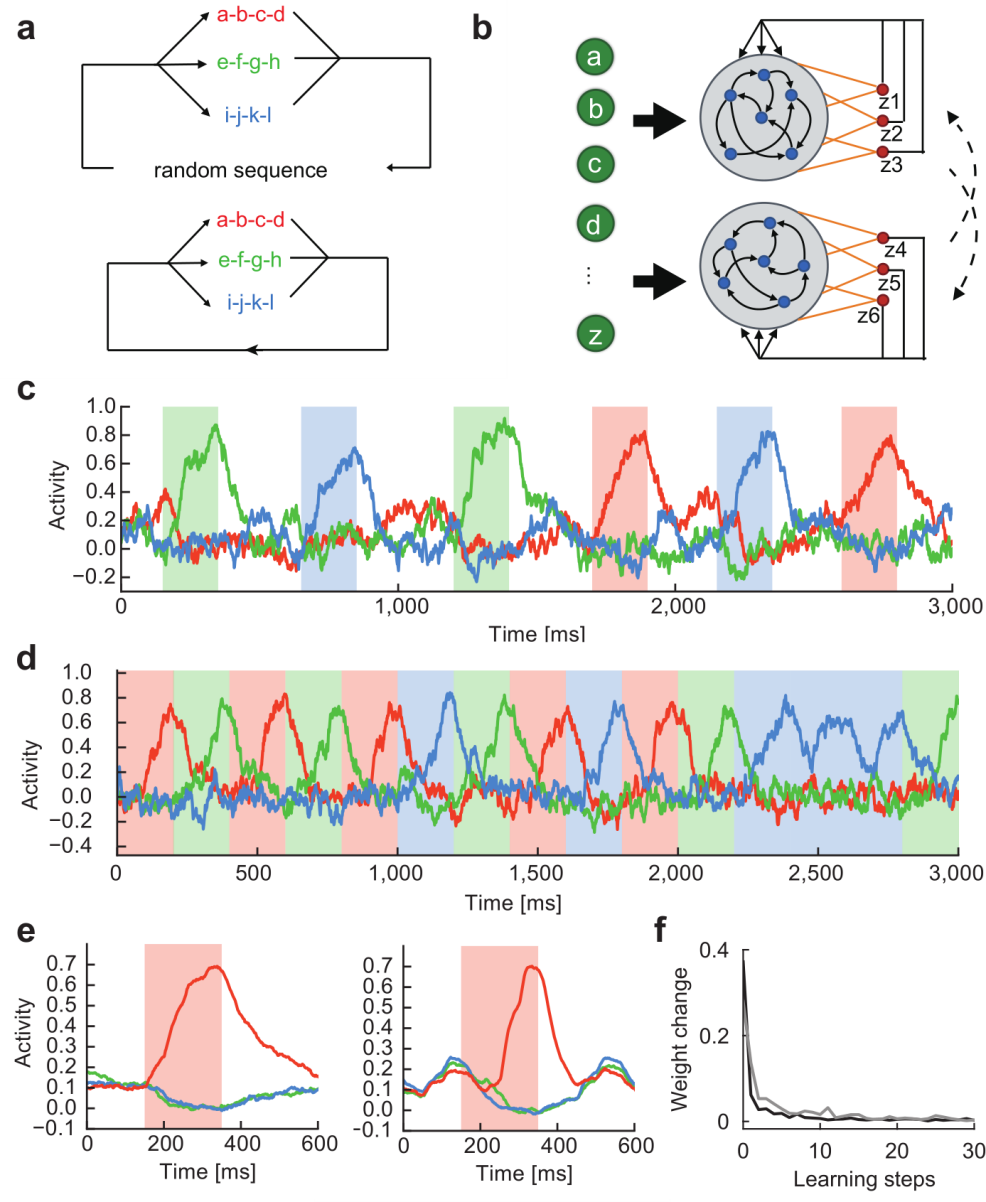
Readout activity after learning detects multiple chunks. **(a)** Top, Three chunks a-b-c-d (red), e-f-g-h (green), and i-j-k-l (blue) separated by random sequences are recurred at equal frequencies in input. Bottom, The three chunks are repeated without the intervals of random sequences. **(b)** Each reservoir was connected to three readout units. **(c)** Selective readout responses to the individual chunks (colored intervals) were self-organized. Input contained random sequences. The responses are colored according to their selectivity to the chunks. **(d)** The same chunks were repeated without breaks by random sequences. Previous models of chunking typically processed such input sequences. **(e)** Readout activities formed with (left) and without (right) random sequence intervals were averaged over the recurrence of chunk “a-b-c-d”. **(f)** Time evolution of average readout weights is shown at every step of learning with (gray) and without (black) random sequence intervals.

We can extend the previous learning rule for learning multiple chunks without difficulty. To show this, we embedded three chunks into a random input sequence (Fig 2A, top). The three chunks had the same occurrence probability of 1/3. To process this complex input sequence, we made two modifications to the previous model. First, each reservoir was connected to three readout units (*z*_1_, *z*_2_, *z*_3_ for the 1st reservoir and *z*_4_, *z*_5_, *z*_6_ for the 2nd reservoir), each responsible for the learning of one of the three chunks (Fig 2B). Second, we modified the teaching signals as follows:

$$f_{a}\left(t\right)={\left[\tanh\left(\left({\hat{z}}_{a\prime }\left(t\right)-\gamma \sum_{c=4,5,6}^{\prime }{\hat{z}}_{c}\left(t\right)\right)/\beta \right)\right]}_{+}\;\left(a=1,2,3\right)$$ (2)

$${f_{b}(t)=[tanh(({\hat{z}}_{b\prime }(t)-\gamma \sum_{c=1,2,3}^{\prime }{\hat{z}}_{c}(t))/\beta )]}_{+}\;(b=4,5,6)$$ (3)

where *a*’ and *b*’ refer to the corresponding readout units of the partner RC modules (i.e., *a*’ = *a*+3 and *b*’ = *b*-3), and dashes in the second term indicate that the corresponding readout unit should be excluded from the summation. The constant *γ* was set as 0.5. Thus, teaching signals were exchanged between the RC modules as in the previous case. Each readout unit receives a triplet of teaching signals from the partner network, in which one is cooperative and the other two are competitive (S2A Fig). These signals allow each readout unit to adopt to a specific chunk, but the chunk to be learned by a readout unit is not a priori specified because the teaching signals are symmetric with respect to the permutation of indices per reservoir. A further extension of the learning rule to an arbitrary number of chunks is straightforward.

As in the case with a single chunk, each readout unit displayed a ramping activity selective to a specific chunk, signaling successful chunk learning (Fig 2C). During this learning, teaching signals also self-organized such that each pair of the readout units eventually exhibited a selective response to a specific chunk, indicating that the teaching signals work adequately (S2B Fig). The complex form of teacher signals looks somewhat biologically unrealistic, but they can easily be implemented by inhibitory neurons (S2C Fig: see Methods) to generate chunk-selective phasic readout responses (S2D Fig). Below, inhibitory neurons are not explicitly modeled.

The question then arises whether the RC system could also learn multiple chunks when they occur continuously without temporal separations by random sequences. To study this, we trained the model by using input sequences in which three chunks appear randomly and consecutively with equal probabilities, without any interval of random sequences (Fig 2A, bottom). Thus, the same RC system as before could easily learn multiple chunks (Fig 2D). A notable difference was that, outside of the chunks, the readout activity decayed faster for undisturbed sequences than for temporally separated ones (Fig 2E). In fact, learning proceeded faster for the former sequences (Fig 2F), suggesting that learning is more effective when chunks are not disrupted by random sequences. Throughout this study, one learning step corresponds to 15 sec.

### Selective recruitment of reservoir neurons for chunk learning

Next, we investigated how the activities of reservoir neurons encode chunks. Here, the network was trained on sequences containing three chunks and random sequences. In each reservoir, a subset of neurons selectively responded to each chunk after learning (S3A Fig). Therefore, we classified reservoir neurons into three ensembles according to the selectivity of their responses to each chunk (Methods). Some reservoir neurons responded to more than one chunk, but they were excluded from the following analysis for the sake of simplicity. Each neural ensemble received slightly stronger inputs from the specific chunk it encoded, which then determines the selectivity of the encoding ensemble (S3B Fig). Through learning, the neural ensemble encoding a particular chunk developed stronger projections to the corresponding readout unit compared with other neural ensembles (S3C Fig). Consistent with this, the distribution of readout weights was more positively skewed in encoding ensembles than in non-encoding ensembles (S3D Fig). Moreover, the readout unit projected back to the corresponding encoding neuron ensemble more strongly than to the other ensembles (S3E Fig). Because feedback connections were not modifiable, these results imply that readout connections were strengthened between readout units and reservoir neurons that happened to receive relatively strong feedback from the readout unit.

### The role of low-dimensional network dynamics in chunk learning

**Fig 3:**
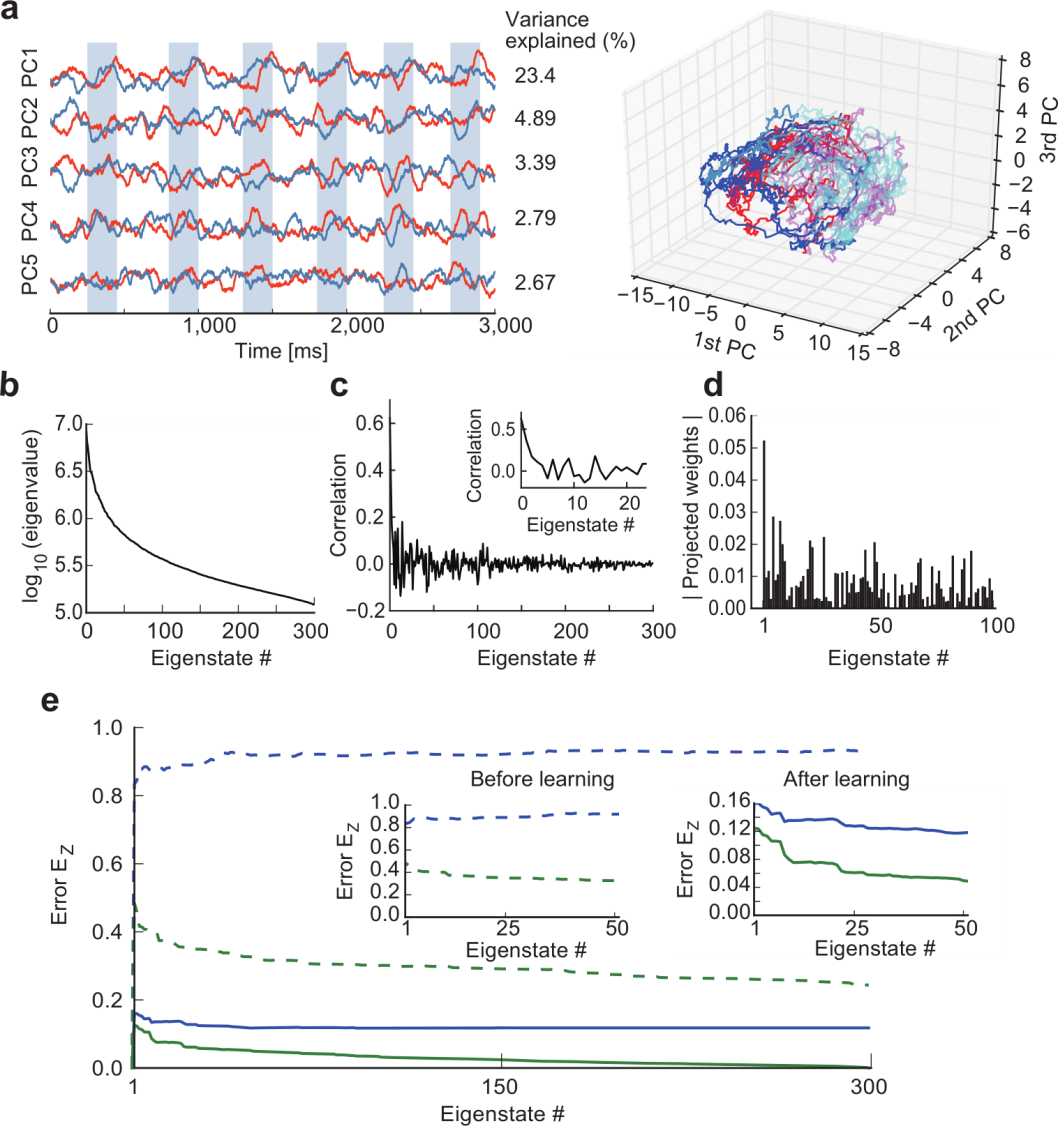
Principal component analysis of recurrent networks. Each recurrent network consists of 300 neurons. **(a)** Left, Activities of two reservoir networks are projected onto the top five eigenvectors of the correlation matrix. Shaded areas indicate the intervals of the presentation of chunks. Numerals on the right side show the variances explained. Right, The low-dimensional trajectories of the two reservoir modules are shown in the space spanned by PC1 to PC3. Red/blue or magenta/cyan portions show trajectories during the epoch of non-vanishing or vanishing teacher signals, respectively. **(b)** The eigenvalues of PCs are shown in a logarithmic scale. **(c)** The correlation coefficient between each PC and the readout activity is shown. **(d)** The length of readout weights projected onto each eigenvector is shown for first 100 eigenstates. **(e)** “Within-self” difference between the R1-output and the projected R1-output (green) and “between-partner” difference between the R2-output and the projected R1-output (blue) are shown for all the eigenstates before (dashed) and after (solid) learning. Insets display magnified versions for major eigenstates.

To gain further insight into the mechanism of chunking, we explored the low-dimensional characteristics of the dynamics of reservoir networks. In our model, the two RC modules, termed R1 and R2, are thought to mimic others. This would be possible when the two recurrent networks receiving the same input sequence predict the responses of other modules well. To see how this prediction is formed, we calculated the principal components (PCs) of the post-learning activity of trained recurrent networks in the example shown in Fig 1. After learning, the lowest principal component (PC1) but not the other PCs, of each reservoir resembled the phasic response of the corresponding readout unit during the presentation of chunks (Fig 3A, left). The learned trajectories wandered in the low-dimensional PC space outside the chunks where teacher signals vanished, while, inside the chunks, non-vanishing teacher signals rapidly constrained both trajectories in narrower regions showing similar PC1 values (Fig 3A, right). This behavior is understandable because the eigenvalues of PCs decay rapidly (Fig 3B). Interestingly, the correlation coefficient between each PC and the readout activity decayed more dramatically (Fig 3C). Accordingly, the direction of readout weight vector was more strongly correlated with that of PC1 compared to other PCs (Fig 3D). These results suggest that the low-dimensional characteristics of neural dynamics play a pivotal role in encoding the chunks.

We then determined to what extent the responses of R1 and R2 are represented by the low-dimensional dynamical characteristics of R1. We calculated the PCs of recurrent network dynamics in R1, and expanded its population rate vector and readout weight vector up to the *M*-th order of these PCs (*M* ≦ *N*_G_). Then, we reconstructed the output of R1 by using the *M*-th order rate vector and the *M*-th order weight vector on the low-dimensional subspace spanned by the first *M* PCs (Methods). In Fig 3E, we calculated the differences between the reconstructed R1-output and the full outputs of R1 (within-self difference) and R2 (between-partner difference). Before learning, both differences remained large as *M* was increased. After learning, the “within-self” difference rapidly decreased for *M* < 30–40 and then gradually approached zero. The “between-partner” difference also rapidly dropped for relatively small values of *M*, but it stopped decreasing for *M* > 50, remaining at relatively large values. These results suggest that R1’s reservoir, as well as R2’s reservoir, learns to mimic the partner's response by using the low-dimensional characteristics of its recurrent neural dynamics.

The role of low-dimensional neural dynamics in a broad range of computation was recently explored in a class of recurrent network models with a minimal connectivity structure [30], which is a combination of a low-rank structured matrix and a random unstructured matrix. The low-rank matrix may be trained to give task-related low-dimensional dynamics whereas the random matrix may generate chaotic fluctuations useful for learning. The RC system can be approximately viewed as such a network, where the product of readout weight vector and feedback weight vector (***J***^*GZ*^)^T^***w*** defines a rank-one matrix and recurrent connections in the reservoir gives a random matrix. It will be intriguing to study the present chunk learning in the theoretical framework.

### Network- and chunk-size dependences of learning

Chunk learning may be easier and more accurate if chunks were shorter or network size is larger. However, we did not find a sharp drop of performance when the size of chunks was increased. To observe this, we first measured learning performance for two chunks with the sizes 4 and 7 by varying the network size. Instantaneous correlations were calculated between the activity of a readout unit and a reference response pattern, which takes the value 1 during the presentation of a chunk and is 0 otherwise, every 15 s during learning and were averaged over 20 independent simulations. Note that the maximum value of the correlation was 0.5 if the readout activity grows linearly from 0 to 1 during the chunk presentation. S4A Fig shows the correlations for input sequences containing the short or long chunk in networks of sizes *N*_*G*_ = 30, 300, and 500. Correlations were nearly zero before learning, but reached similar maximum values approximately within ten steps of learning. The average value of the correlations was generally larger for chunk size 4 than for chunk size 7, but the differences were not significant (S4B Fig).

Second, we measured learning performance by varying the size of chunks with the network size fixed (*N*_G_ = 300). In this simulation, we alternately presented a single chunk with the size *s* and random sequences of the sizes *s* + 2 to *s* + 5, where each element of the random sequences was chosen from a set of 4*s* elements. Thus, the dual RC system had 5*s* input neurons. When the chunk size exceeded 10 (S4C Fig), the value of correlation rapidly dropped, suggesting the existence of a critical chunk size beyond which learning performance is degraded. For *s* = 4, learning performance showed unexpectedly large fluctuations due to some unknown reason. The explicit evaluation of the critical chunk size requires an analytic approach, which is beyond the scope of this study.

In addition, a larger network did not necessarily show better performance. The magnitude of the post-learning instantaneous correlation was not significantly increased when the network size was 200 or greater (S4B Fig). Thus, the performance of chunk learning does not scale with the network size. This is not so surprising because increasing the size of the reservoirs does not necessarily increase the variety of neural responses useful for learning if the size is already sufficiently large. This seems to be particularly the case in the proposed mechanism because it heavily relies on the low-dimensional characteristics of neural dynamics (S3A Fig).

### Crucial role of noise in chunk learning

**Fig 4:**
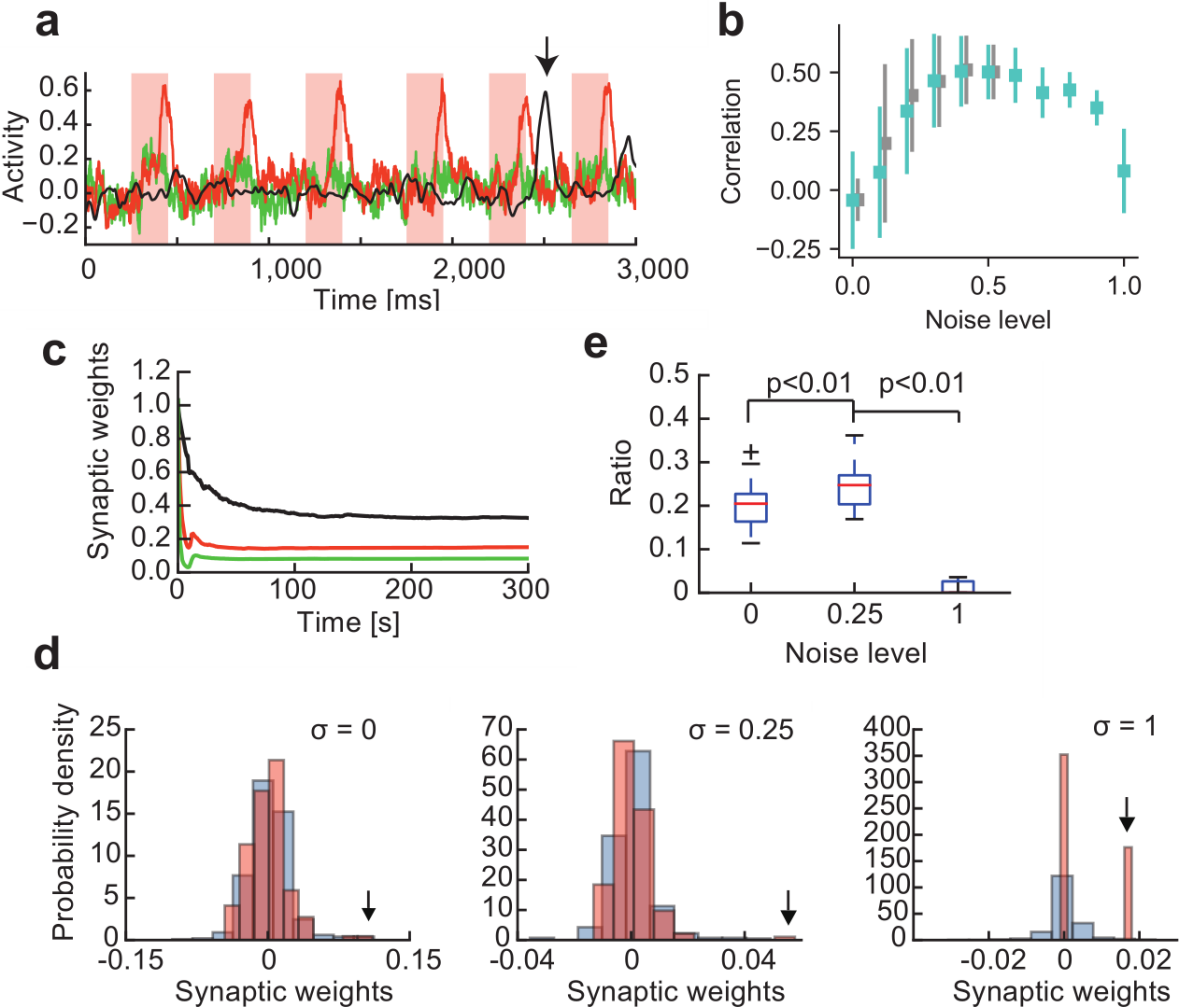
Effects of noise on successful chunk learning. **(a)** Activity of a readout unit after learning a chunk at different noise levels: *σ* = 0 (black), 0.25 (red) and 1 (green). Without noise, the readout unit still learned to respond to a portion of input, but this portion did not necessarily belong to a chunk (vertical arrow). **(b)** Learning performance is a non-monotonic function of the noise level. The optimal performance was obtained at *σ* = 0.4–0.6 when the scaling factor in Equation 4 was set as *g*_G_ = 1.5 (cyan). The effect of noise on the learning performance was not significantly changed when the scaling factor was simultaneously reduced with the noise level (gray). **(c)** Evolution of the norm of readout weights during learning is shown for *σ* = 0 (black), 0.25 (red) and 1 (green). **(d)** The distributions of readout weights from chunk-encoding (red) and non-encoding (blue) reservoir neurons are shown after learning at different noise levels. Arrows indicate the maximum weight values from the chunk-encoding neurons. **(e)** The fraction of strong readout weights (see the main text) from the encoding neurons is shown for different noise levels. The fraction is significantly larger for *σ* = 0.25 compared with *σ* = 0 and 1 (p<0.01, Mann–Whitney U test).

We found that external noise plays an active role in successful chunking. We demonstrated this in the case where the input only contained a single chunk (Fig 4A). In the absence of noise readout units, phasic responses were still observed, but these responses were not necessarily time-locked to chunks (Fig 4A, vertical arrow). As shown later, the two RC modules in principle may agree on an arbitrary feature of the input sequence, which implies the RC system may converge to a local minimum of learning. Noise may help the system to escape from the local minima. On the other hand, too strong of noise completely deteriorated the phasic responses to chunks. Thus, the RC system could learn chunks only when a modest amount of external noise existed (Fig 4B). In the presence of adequate noise (*σ* = 0.25), the average weight of the readout connections rapidly decreased to a small equilibrium value during learning (Fig 4C), leaving some readout weights much stronger than the majority (Fig 4D). This reduction was expected because external noise gives a regularization effect on synaptic weights in error-minimization learning [31]. The strong weights were obtained for readout connections from the reservoir neurons responding to the chunk, hence they were crucial for chunk detection. However, this was not the case in the absence of noise (*σ* = 0). We counted the fraction of strong readout connections that emerged from chunk-encoding reservoir neurons, where strong connections included those that were greater than the standard deviation of the weight distribution. Such a fraction was significantly larger in the presence of adequate noise than in the absence of noise. Under strong noise (σ = 1), although the weight distribution becomes more bimodal, the noise disrupted learning and the system failed to capture the chunks (Fig 4E).

Another possible mechanism in which the external noise would improve the learning performance is that the dynamics of RC modules with weak noise are too far in the chaotic regime and the external noise suppresses chaos to enable proper chunk learning [32]. To test this possibility, we compensated a decrease of *σ* by decreasing the strength of recurrent conections *g*_*G*_, which weakens the influences of chaos, and investigated whether the deterioration of performance is suppressed. The noise intensity was decreased from a modest level (*σ* = 0.5), and the values of *σ* and *g*_*G*_ were decreased at the same rate. Although the improvement was not significant, the dual RC system better resisted the performance deterioration (Fig 4B), suggesting that proper chunk learning requires a certain balance between external noise and chaotic dynamics.

Though our results so far suggest that mutual supervision enables the RC system to learn recurring groups of items in a sequence, these results do not indicate how the system chooses particular groups for learning. The question then arises whether our model detects a “chunk” if a sequence merely repeats each letter randomly without temporal grouping. To study this, we constructed a set of input sequences of ten letters, where all the letters appeared equally often in each sequence. We then exposed the RC system with a readout unit to these sequences. We found that the system learned to respond to one of the letters with approximately equal probabilities (S5A Fig). We then made the occurrence probability of letter “a” twice as large as the occurrence probabilities of the others and found that the system detected “a” about twice as frequent as the others (S5B Fig). These results indicate that the learning performance of the dual RC system relies on the occurrence frequency of repeated features if there are no other characteristic temporal features in the input sequence.

The frequency dependence of our model partially accounts for the features of sequences that are grouped into chunks. As demonstrated in Fig 3, a pair of RC modules engage in the mutual prediction of the partners' response. This prediction would be easier for the items in the input that repeatedly occur in a fixed temporal order. However, the explicit role of temporal grouping in chunking remains to be further clarified.

**Fig 5:**
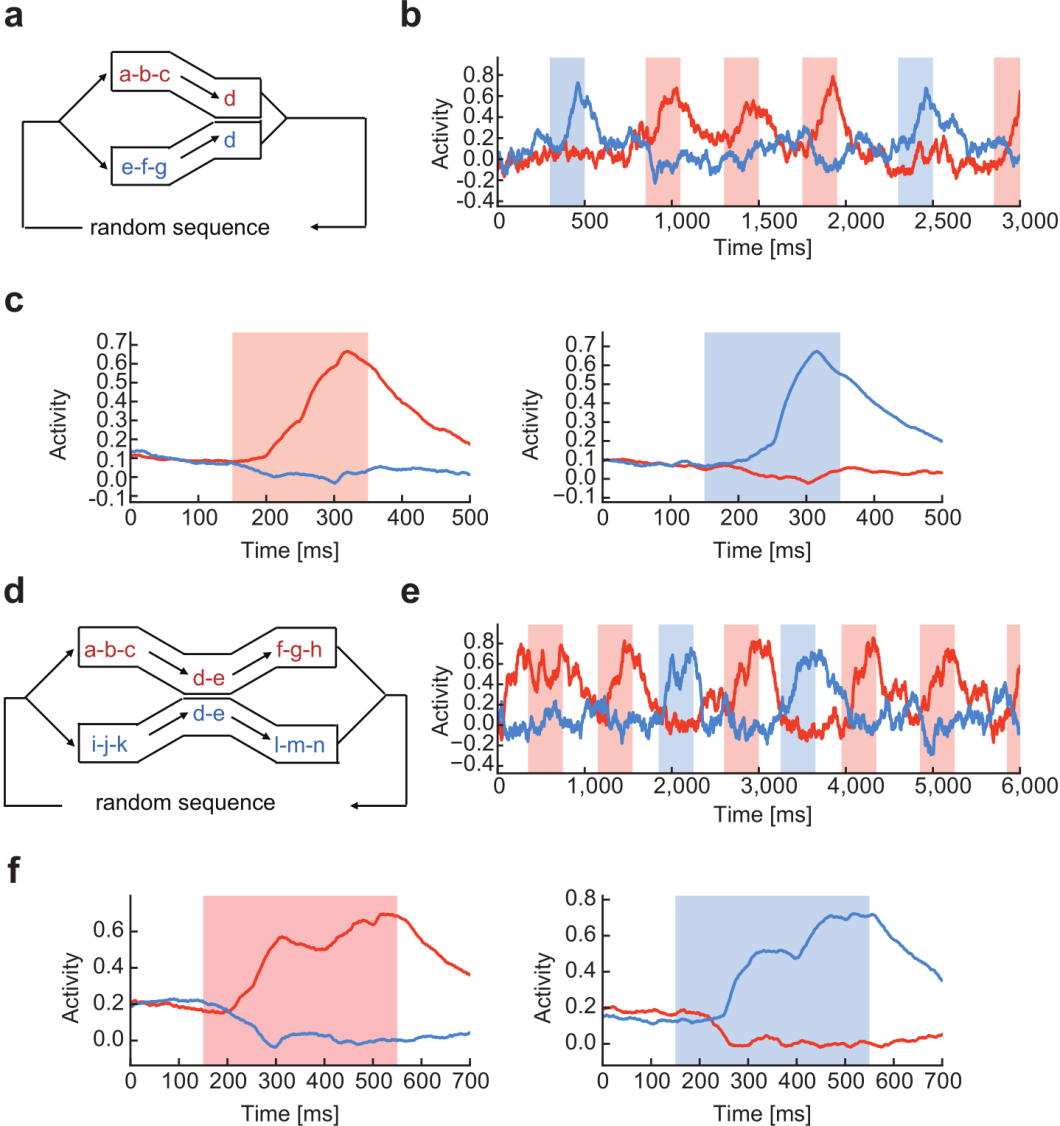
Learning chunks with mutual overlaps. **(a)** Two chunks shared the last component “d” in a random input sequence. **(b)** Activities of two readout units were selective to different chunks after learning. **(c)** The average response profiles are shown for the two readout units. **(d)** Two chunks shared the middle components “d-e” in a random input sequence. **(e)** and **(f),** Activities of two readout units and the average response profiles are shown, respectively.

We then demonstrate that the RC system can simultaneously chunk multiple sequences with overlaps, where input sequences share some letters as common items. In some sequences, common subsequences appeared in the beginning or the end of chunks (Fig 5A), whereas other sequences involved common subsequences in the middle of chunks (Fig 5D). In both cases, the RC system (with two readout units) successfully chunked these input sequences without difficulty (Fig 5B and 5E). Interestingly, the activity of the readout units averaged over repetitive presentations ceased to increase during the presentation of the overlapping part of the chunks (Fig 5C and 5F). This seems reasonable as overlapping in part does not contribute to the prediction of the following items in the chunks and hence needs not be learned.

### Chunking sequences of realistic inputs

**Fig 6:**
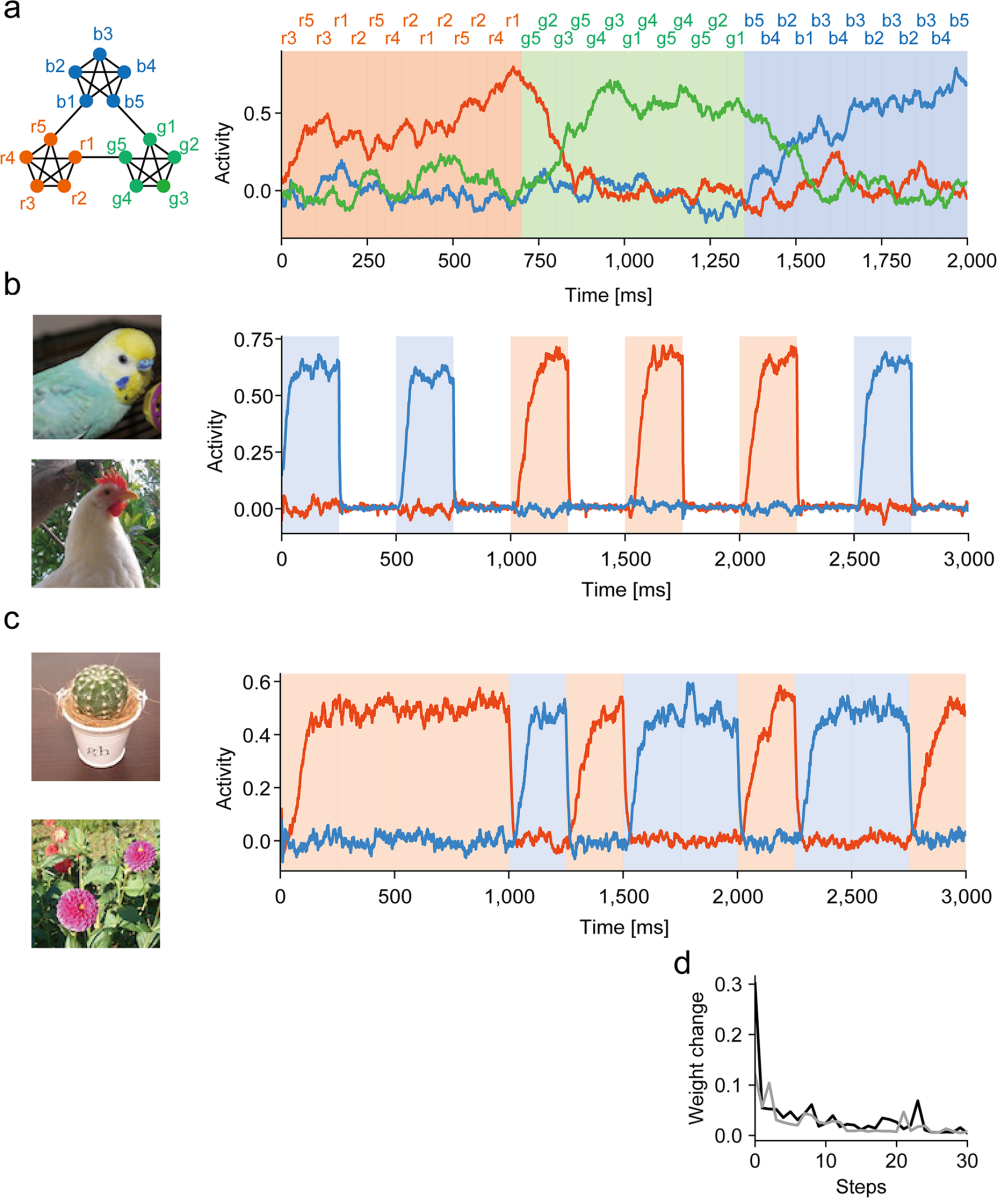
Chunking complex temporal inputs. (a) Sequence inputs were generated by a graph with uniform transition probabilities and community structure. The graph was modified from [23]. (b) Sequence of high -resolution (97x97x3) visual stimuli, where the factor 3 represents the three RGB channels, was chunked. White intervals show periods of Gaussian noise. (c) Sequence of high-resolution (97x97x3) visual stimuli was chunked. (d) Learning curves are compared for the images shown in (c) between high (black) and low (gray) resolution versions. The images were repeatedly presented without noise intervals.

So far, we have only studied discrete sequences of letters with varying complexity. However, the applicability of the proposed mechanism is not restricted to this relatively simple class of temporal inputs. We first showed the potential advantage of this mechanism over the conventional statistical methods, considering a system with three readout units (per RC module) for processing sequence inputs generated by a random walk through a graph (Fig 6A). This was previously used in examining the learning ability of event recognition by human subjects [23]. Each node of this graph has exactly four neighbors, and hence is visited by random walk with uniform transition probabilities over all neighbors. Despite this uniformity, the graph has three clusters of densely connected nodes, which define the communities in the graph [33, 34]. Human subjects and our model (Fig 6A) can easily chunk these clusters according to community structure, but machine-learning algorithms based on surprise signals (e.g., [21]) cannot [23].

We further demonstrated that the proposed system can learn to detect two images recurring in visual input streams with (Fig 6B) and without (Fig 6C) random intervals of Gaussian noise stimuli. We examined whether learning speed depends on the resolution of images and found that such a dependence was weak if the network size was unchanged (Fig 6D). Our results show the potential ability of the proposed mechanism in analyzing the community structure of a broad class of temporal inputs.

## Discussion

Conventional statistical methods of chunking use unequal transition probabilities between sequence elements as cues for sequence segmentation. In contrast, we propose a conceptually novel framework in which the neural system self-organizes its internal dynamics to respond preferentially to chunks (i.e., frequently recurring segments) with a temporal input, rather than attempts to predict the temporal patterns of input sequences. We achieved this unsupervised learning in a network of paired RC modules mutually learning the responses of the partners. Sequence leaning with RC has been studied in motor control [35–37] and decision making [38, 39]. Theoretical extensions to spiking neuron networks [40] and/or reward-based learning [41] have thus been proposed. In this study, we showed that RC can be used for the unsupervised learning of hidden structure of continuous information streams.

Chunking has often been accounted for by predictive uncertainty or surprise [17, 42–44]. However, recent evidence suggests the existence of an alternative mechanism of chunking in which events are segmented based on the temporal community structure of sequential stimuli [23]. Indeed, it has been shown that individual items in a sequence are concatenated into an event when they frequently go together in the sequence. This dual RC system automatically chunks a continuous flow of stimuli based on temporal clustering structure and the occurrence probabilities of the stimuli without relying on predictive uncertainty or surprise. In addition, the model can chunk clusters of sequence elements that cannot be chunked by conventional statistical methods based on unequal transition probabilities (Fig 6A). Unsupervised chunk learning was previously modeled by using heteroclinic orbits in a dynamical neural system [19]. Though this mechanism enables the learning of prescribed chunks, whether it also offers flexible learning of arbitrary chunks remains unclear.

Our model has some advantages over the previous models of chunking. Our model can detect multiple chunks embedded into random background sequences. To our knowledge, the detection of multiple chunks has not been seriously attempted in the presence of various types of input noise on chunking. Further, as shown in Fig 5 our model can learn multiple partially overlapping chunks without additional mechanisms, which was also previously difficult. On the other hand, a weak point is that our model requires specially designed teaching signals, which depend on the structure of chunks. Related to this, mutually inhibitory teaching signals were introduced in an ad-hoc manner to prevent multiple readout units from learning the same chunk. A more flexible mechanism of learning should be further explored.

The dual RC system described here shows good performance in the presence of external noise. Without noise, the system also learns to respond to certain segments of a sequence, but these segments may not coincide with any of the frequently repeated chunks. An adequate amount of external noise eliminates such spurious responses and enables the system to respond to the most prominent features of a sequence, namely repeated chunks. This finding is interesting because the initial state of the dual RC system is chosen on the so-called “edge of chaos,” on which weakly chaotic neural dynamics provide an adequate amount of flexibility for supervised learning [29, 45–46]. Moreover, the present system assumes a similar initial state, but additionally requires the regularization of synaptic weight dynamics by noise (Fig 4C). Training a recurrent neural network with an explicit regularization term is known to eliminate the strange neuronal responses that are not observed in the motor cortex [37].

The dual RC system learns sequence in an unsupervised fashion by using two neural networks and, in this sense, is similar to Generative Adversarial Network (GAN) in deep learning [47]. A critical difference, however, exists between the two models. In GANs, a generative network learns to mimic the structure of training data and a discriminative network learns to distinguish between samples from the training data and those generated by the generative network. Because the generative model learns to deceive the discriminative model, GANs learn the structure of data distribution under a conflicting cost function. By contrast, in the dual RC system, two neural networks learn to help each other for the formation of a consensus about the structure of temporal inputs. Therefore, our model is conceptually different from GANs.

The structure of our model has an interesting similarity to cortico-basal ganglia loops, where two reservoirs may represent bi-hemispheric cortical networks and readout units may correspond to striatal neurons. The responses of readout units and those of striatal neurons in the formation of motor habits also look similar. Sequential motor behavior becomes more rigid and automatic over the course of learning and practice, and the basal ganglia is thought to play a pivotal role in habit formation [9, 48]. For instance, in rats running in a T maze, the majority of dorsolateral striatal neurons exhibit burst firing when the run is initiated or completed, or both [49]. Similarly, in mice an increased population of striatal neurons selectively responds to the initial (Start cells), the last (Stop cells), or both actions in the trained behavioral sequence [7, 8]. In our model, readout units respond strongly to the last component of each chunk, similar to the Stop cells. Our model predicts that the Stop-cell’s response may decrease when two motor chunks have overlapping portions (Fig 5). On the other hand, our model does not show Start cell-like responses. Whether and how Start cells are formed is an intriguing open question.

The proposed learning scheme works most efficiently when two RC modules are not interconnected, but rather work independently. In fact, the performance of chunk learning drops below 50% of the initial level when the connection probability between the two reservoirs exceeds about 10% (S4D Fig, see the Methods). This suggests that each RC module can obtain maximum information about temporal input when it receives the teaching signal completely from its outside. The existence of inter-reservoir connections implies that some portion of the teaching signal originates from its inside. Where can such independent networks be located in the brain? One possibility is that they are represented by mutually disconnected recurrent neuronal networks in a local cortical area. Because they are functionally equivalent, it is unlikely that they are implemented in functionally distinct areas. Another intriguing possibility is that they are distributed to functionally equivalent cortical areas in different hemispheres. Indeed, the inferior frontal gyrus and the anterior insula are bilaterally activated when human subjects chunk visual information streams [23, 50]. Whether subnetworks of pyramidal cells perform chunking in these or other cortical areas [51] remains an intriguing open question.

In summary, we propose an unsupervised learning system by combining two independent reservoir computing modules. During learning, the two systems supervise each other to generate coincident outputs, which in turn allows the entire system to consistently learn chunks hidden in irregular input sequences. As chunking is a fundamental step in the analysis of sequence information, our results have significant implications for understanding how the brain models the external world.

## Methods

### Details of the neural network models

In this study, the proposed model is composed of two recurrent networks (reservoirs). Each recurrent network is composed of *N*_*G*_ neurons. Each neuron follows the following dynamics as *i* = 1,2,… *N*_*G*_,

$$\tau \dot{x_{i}}(t)=-x_{i}(t)+g_{G}\;\sum_{j=1}^{N_{G}}J_{ij}^{GG}r_{j}(t)+J_{i}^{GZ}z(t)+\sum_{\mu =1}^{N_{I}}J_{i\mu }^{GI}I_{\mu }(t)+\sigma \xi_{i}(t),$$ (4)

$$r_{i}(t)=\tanh\left(x_{i}(t)\right),$$ (5)

where *I*_*μ*_(*t*) is the activity of input neurons, *ξ*_*i*_(*t*) is a random (Wiener) process and *σ* is the standard deviation. *N*_*I*_ is the number of input neurons. The parameter *g*_*G*_ determines the complexity of the behavior of the reservoir, and shows chaotic spontaneous activity if *g*_*G*_ > 1. The instantaneous output is given by *z*(*t*) = ***w***^T^***r***(*t*), where ***w*** is the readout weight vector. The readout unit is connected with *n* reservoir neurons by the readout weights ***w***. The readout weights are modified according to the FORCE learning rule in which the error between the actual output and the teaching signal is minimized [29]. The activity of the readout unit is transmitted to the reservoir via the feedback.

The initial values of the readout weights ***w*** are generated by a Gaussian distribution with the mean 0 and variance 1/*n*. Each element of the feedback coupling *J*^*Gz*^ is randomly sampled from a uniform distribution [-1, +1]. In the connection matrix *J*^*GG*^ of the reservoir, each element is taken from a Gaussian distribution with mean 0 and variance 1/(*pN*_*G*_), where *p* is the connection probability of the reservoir neurons. In the connection matrix *J*^*GI*^ between input neurons and the reservoir, each row has only one non-zero element drawn from a normal distribution of mean 0 and variance 1. We simulated the model with time steps of 1 [ms].

The values of parameters used in simulations are as follows: in Figs 1, 3 and 4 and S1 Fig, S4C Fig, S4D Fig, and S5 Fig, *N*_G_ = 300,*p* = 1,*n* = 300 and *σ* = 0.3; in Figs 2 and 6, S2 Fig, and S3 Fig, *N*_G_ = 600,*p* = 0.5,*n* = 300 and *σ* = 0.1; in S4A Fig and S4B Fig, *p* = 1,*σ* = 0.3 and *n* = *N*_G_ while the values of *N*_G_ were varied; in Fig 5, *p* = 1,*n* = 300, and *N*_G_ = 800,*σ* = 0.15 (b) or *N*_G_ = 500,*σ* = 0.1 (e). The number of input neurons was *N*_I_ = 26 in all simulations except S4C Fig, in which *N*_I_ was 5*s* with *s* being the size of the chunk. In all simulations, *τ* = 10 [ms] and *g*_G_ = 1.5. The learning rate was set as *α* = 100 because larger values could cause instability in the learning process. The network was trained typically for several hundreds of seconds except in Figs 2, 5B and 5E where the simulation time was 5000, 2500 and 25,000 [s], respectively.

### Teaching signals mediated by interneurons

In S2D Fig, the teaching signals were generated as

$${f_{a}(t)=[tanh(({\hat{z}}_{a\prime }(t)-\gamma \sum_{c=4,5,6}^{\prime }y_{c}(t))/\beta )]}_{+}\;(a=1,2,3),$$ (6)

where the activities of interneurons were calculated as

$$\tau \dot{y_{c}}(t)=-y_{c}(t)+{\hat{z}}_{c}(t).$$ (7)

A similar formula applied to the partner network. Note that a dash in the second term of Eq 6 indicates that the corresponding readout unit should be excluded from the summation (S2C Fig).

### Connections between the reservoirs

In S4D Fig, the weights of recurrent connections in each reservoir module and those of connections between the modules were sampled from an identical Gaussian distribution with mean 0 and variance 1/{(1 + *q*)*N*_*G*_}, where *q* is the connection probability of inter-module connections. The recurrent connections were all-to-all. The value 1 in the denominator was introduced such that the limit *q* → 0 gives the disconnected RC modules studied in other panels in S4 Fig.

### Normalized output for teaching signals

In our learning rule, we changed the outputs of readout units such that the mean outputs coincide with zero and the standard deviation becomes unity:

$$z(t)⟶\hat{z}(t)=\frac{z(t)-\mu (t)}{\sigma (t)},$$ (8)

where *μ*(*t*) and *σ*(*t*) were calculated as

$$\mu (t)=\frac{1}{T}\;\int_{t-T}^{t}z(t^{\prime })dt^{\prime },$$ (9)

$$\sigma (t)=\sqrt{\frac{1}{T}\int_{t-T}^{t}{z(t^{\prime })}^{2}dt^{\prime }-{\mu (t)}^{2}},$$ (10)

with a sufficiently long period *T* (= 15 [s]). The modified output $\hat{z}(t)$ was then transformed by two nonlinear functions to generate the teaching signal shown in the Results.

### Selectivity of reservoir neurons

In S3A Fig, the activities of all reservoir neurons were first averaged and then normalized. To define the response selectivity of neurons, we sorted all of the neurons by their mean activation phases defined as,

$${\hat{t}}_{i}=\frac{T}{\pi }\arg\left[\frac{\sum_{t^{\prime }=1}^{T}{\bar{r}}_{i}(t^{\prime })\exp\left(i\frac{{2\pi t}^{\prime }}{T}\right)}{\sum_{t^{\prime }=1}^{T}{\bar{r}}_{i}(t^{\prime })}\right]\;[ms],$$ (11)

where $\bar{r}(t)$ is the normalized average response of each cell and *T* = 2400 [ms]. Each reservoir neuron generally showed a significantly large and prolonged phasic response to a particular chunk, which determined the selectivity of the reservoir neuron. We defined a phasic response as such transient activity that exceeded the threshold value μ + 3σ for more than 100 [ms], where μ and σ stand for the average and standard deviation of its activity during the presentation of input sequence. Neurons that were not related to any chunks or responded to multiple chunks were discarded in the analysis.

### Analysis of the low-dimensional dynamics of reservoirs

In Fig 3, we projected the neural responses ***r***_R1_(*t*) of recurrent network in R1 onto the *M* (≦*N*_*G*_) dimensional subspace:

$$r_{R1,M}(t)=V_{M}^{T}r_{R1}(t).$$ (12)

Here, the (*N*_*G*_×*M*)-dimensional matrix ***V***_*M*_ is defined as ***V***_*M*_ = (***φ***_*1*_^(R1)^ ***φ***_*2*_^(R1)^ …***φ***_*M*_^(R1)^) in terms of the *λ-*th eigenvector of R1 reservoir ***φ***_*λ*_^(R1)^. Similarly, we projected the readout weight vectors from R1 onto the same subspace as

$$w_{R1,M}=V_{M}^{T}w_{R1}.$$ (13)

We then calculated the difference between the actual output of R1 and the output reconstructed on the subspace as

$$E_{z}=\sqrt{\frac{1}{T}\int_{0}^{T}{\left|z_{R1}(t)-w_{R1,M}^{T}r_{R1,M}(t)\right|}^{2}dt}.$$ (14)

The difference between the actual output of R2 and the projected R1-output was calculated in a similar fashion.

### Simulations of visual information streams

In Fig 6B and 6C, we constructed a pair of RC modules each having two readout units. A stream of two images with high (97x97 pixels x 3 RGB channels) or low (32x32 pixels x 3 RGB channels) resolutions was used as input, in which the presentations of two images (and Gaussian noise images in Fig 6B) were randomly switched at every 250 ms. Each reservoir neuron received input from randomly chosen 10% of pixels. In Fig 6D, the low-resolution versions of the images used in Fig 6C were created at the reduced size of 32 x32 pixels (x 3 RGB channels).

All codes for computer simulations were written in Python 3 and are available at https://github.com/ToshitakeAsabuki/dualRC_codes.

## Supporting information

S1 Fig Responses of readout neurons during learning procedure. Below, numerical results are shown for the model simulated in Fig 1. The responses of two readout neurons were initially incoherent (top). They gradually developed strong coherent responses to the repetition of a chunk as learning proceeded (middle and bottom).
(EPS)

S2 Fig Structure of teaching signals for multiple chunk learning. **(a)** A schematic illustration for the structure of teaching signals for *z*_1_. Since the partner node of *z*_1_ is *z*_4,_ the sign of the corresponding term in the teaching signal is positive whereas the other terms are negative. **(b)** Teaching signals show incoherent activities (top) before learning, while the learning procedure makes these activities much coherent (middle and bottom). Thick and thin lines represent teaching signals from the pair of the readouts. **(c)** Lateral inhibitions by interneurons are modeled. **(d)** The activities of readouts after learning with interneurons.
(EPS)

S3 Fig Cell assemblies selected in the reservoirs. **(a)** The activity of each reservoir neuron was averaged over repeated trials and normalized by its maximum activity. Neurons were sorted according to the onset times of their activations to reveal the cell assemblies encoding the three chunks (Methods). **(b)** The distributions of input weights onto each cell assembly are shown for input neurons belonging to the corresponding chunk (solid) and the others (dashed). The solid and dashed distributions summed over all cell assemblies were significantly different (p = 0.011, t-test). **(c)** Temporal evolution is shown for average weights from encoding cell assemblies to the corresponding readout units. **(d)** Normalized distributions are shown for readout weights from each cell assembly. **(e)** The distribution of feedback weights from readout units to each cell assembly is shown.
(EPS)

S4 Fig Learning with different sizes of reservoirs and chunks. **(a)** The time course and performance of learning are shown for an input sequence involving a single chunk of the length 4 (blue) or 7 (red). Three networks with different sizes (*N*_G_ = 30, 300, 500) were tested. **(b)** The values of the correlation after learning are plotted as a function of the network size. **(c)** The dependence of the correlation on the chunk size is shown. **(d)** The dependence of the correlation on the connection probability between the two reservoirs is presented.
(EPS)

S5 Fig Learning random sequences of single characters. The model shown in Fig 1 was exposed to random sequences consisting of 10 characters (a, b, …, j). Input sequences had no apparent temporally grouped subsequences. (a) The counts of simulation trials in which each character was learned. All characters appeared equally often in input sequences. In total, 300 trials were performed. (b) Similar trial counts were taken when character “a” appeared twice as often as others.
(EPS)
